# Exosomes derived from smooth muscle cells ameliorate diabetes‐induced erectile dysfunction by inhibiting fibrosis and modulating the NO/cGMP pathway

**DOI:** 10.1111/jcmm.15946

**Published:** 2020-10-03

**Authors:** Jingyu Song, Taotao Sun, Zhe Tang, Yajun Ruan, Kang Liu, Ke Rao, Ruzhu Lan, Shaogang Wang, Tao Wang, Jihong Liu

**Affiliations:** ^1^ Department of Urology and Institute of Urology Tongji Hospital Tongji Medical College Huazhong University of Science and Technology Hubei China

**Keywords:** diabetes, erectile dysfunction, exosomes, fibrosis, NO/cGMP signalling pathway

## Abstract

Erectile dysfunction (ED) is a major health issue among men with diabetes, and ED induced by diabetes mellitus (DMED) is particularly difficult to treat. Therefore, novel therapeutic approaches for the treatment of DMED are urgently needed. Exosomes, nanosized particles involved in many physiological and pathological processes, may become a promising tool for DMED treatment. In this study, we investigated the therapeutic effect of exosomes derived from corpus cavernosum smooth muscle cells (CCSMC‐EXOs) on erectile function in a rat model of diabetes and compared their effect with that of exosomes derived from mesenchymal stem cells (MSC‐EXOs). We incubated labelled CCSMC‐EXOs and MSC‐EXOs with CCSMCs and then observed uptake of the exosomes at different time points using laser confocal microscopy. CCSMC‐EXOs were more easily taken up by CCSMCs. The peak concentration and retention time of labelled CCSMC‐EXOs and MSC‐EXOs in the corpus cavernosum of DMED rats after intracavernous injection were compared by in vivo imaging techniques. Intracavernous injection of CCSMC‐EXOs was associated with a relatively high peak concentration and long retention time. Our data showed that CCSMC‐EXOs could improve erectile function in DMED rats. Meanwhile, CCSMC‐EXOs could exert antifibrotic effects by increasing the smooth muscle content and reducing collagen deposition. CCSMC‐EXOs also increased the expression of eNOS and nNOS, followed by increased levels of NO and cGMP. These findings initially identify the possible role of CCSMC‐EXOs in ameliorating DMED through inhibiting corporal fibrosis and modulating the NO/cGMP signalling pathway, providing a theoretical basis for a breakthrough in the treatment of DMED.

## 1 INTRODUCTION

As one of the most common male sexual dysfunctions, erectile dysfunction (ED) is a common disease in diabetic males. According to epidemiology, the incidence of ED in diabetic patients exceeds 50%,^1^ and by 2045, the number of diabetic patients will reach 629 million.^2^ Penile erection is a complete hemodynamic process that includes corpus cavernosum smooth muscle relaxation, increased arterial blood flow and blocked venous return.^3^ Many studies have shown that important features of ED induced by diabetes mellitus (DMED) are the reduction in corpus cavernosum smooth muscle and decrease in smooth muscle relaxation function under high‐glucose conditions, which leads to an inability to obtain or maintain a satisfactory erection during intercourse.^3^, ^4^, ^5^ DMED is particularly difficult to treat, and phosphodiesterase type 5 inhibitor, a first‐line drug for the treatment of ED, has poor efficacy in DMED.^6^ Therefore, a novel therapeutic approach is needed.

Exosomes are nanosized (40‐150 nm) particles secreted by multiple cell types under physiological and pathological conditions. As an important method of communication between cells, exosomes can carry a variety of substances, such as proteins, lipids and RNAs.^7^ At present, there have been few studies on the treatment of ED with exosomes and even fewer studies on the application of exosomes to DMED. These limited studies have applied only exosomes derived from stem cells such as mesenchymal stem cells (MSC‐EXOs) to rat models of DMED.^8^, ^9^ Therefore, many uncertainties about the therapeutic effect of exosomes on DMED remain. Meanwhile, because the corpus cavernosum is a cavernous tissue with rich blood flow, the ability of MSC‐EXOs to remain in the corpus cavernosum and the ability of cells in the corpus cavernosum to effectively take up MSC‐EXOs may also affect the therapeutic effect of exosomes on DMED, but there has been no relevant research on these topics.

Corpus cavernosum smooth muscle cells (CCSMCs) are important cells distributed throughout the corpus cavernosum that are directly involved in the erection process. As mentioned above, a reduction in the number of CCSMCs and the dysfunction of CCSMCs are important factors in the pathogenesis of DMED. Nevertheless, CCSMCs are unlikely to be directly transferred into the corpus cavernosum. Therefore, we propose a hypothesis about whether DMED can be alleviated by supplementation with exosomes derived from CCSMCs (CCSMC‐EXOs). At present, studies have shown that exosomes derived from vascular smooth muscle cells play important roles in intercellular communication and vascular protection.^10^, ^11^ In addition, in recent years, studies have reported that exosomes derived from vascular smooth muscle can be targeted for enrichment in vascular smooth muscle.^12^ These studies may partly support our hypothesis. Therefore, the aims of this study were to determine the potentially important role of CCSMC‐EXOs in erectile function in a rat model of diabetes by their comparison with MSC‐EXOs and to develop a novel exosome‐based therapy for DMED.

## 2 MATERIALS AND METHODS

### 2.1 Animals

Eight‐week‐old male Sprague‐Dawley rats were acquired from the Laboratory Animal Center of Tongji Medical College, Wuhan, China. This rat model is a classic model used to study DM.^13^, ^14^ We used 10 rats for cell isolation and another 60 rats for animal experiments. Two animals were housed in a rat cage maintained in a controlled environment with a 12‐h light/12‐h dark cycle and had access to standard chow and water ad libitum. Their care and treatment were approved by the Animal Care and Use Committee of Tongji Hospital, Tongji Medical College, Huazhong University of Science and Technology, Hubei, China.

### 2.2 Cell culture and identification

Adipose‐derived stem cells (ADSCs) were isolated from inguinal adipose tissue as described in a previous study.^15^ Briefly, adipose tissue was isolated, minced into small pieces and incubated with 0.075% collagenase type I (Sigma‐Aldrich, St. Louis, USA) for 90 minutes at 37°C. After centrifugation at 1000 × g for 10 minutes, the supernatant was removed. The resulting pellet was treated with erythrocyte lysis buffer for 10 minutes on ice to remove red blood cells. Subsequently, the remaining cells were suspended in Dulbecco's modified Eagle's medium (DMEM) supplemented with 10% foetal bovine serum (FBS), filtered through a 75‐μm mesh strainer and cultured in a 5% CO_2_ incubator at 37°C.

Bone mesenchymal stem cells (BMSCs) were isolated from rat femora as described in a previous study.^16^ Briefly, after the femora were isolated, we used a needle to drill a hole in the distal end of each femur and injected a small volume of serum‐containing medium into the bone to flush the marrow into a dish. Subsequently, the cell suspension was centrifuged at 450 × g for 5 min, and the supernatant was removed. The remaining cells were suspended in serum‐containing medium and cultured in a 5% CO_2_ incubator at 37°C.

After 4 passages, the MSCs (ADSCs and BMSCs) were identified by flow cytometry. Antibodies against the following were used for verification of surface markers: CD29, CD34, CD45 and CD90 (BD Biosciences, San Diego, CA, USA). The multipotency of the MSCs was tested by determining their ability to differentiate into osteoblasts and adipocytes as described previously.^15^, ^17^ Briefly, MSCs were cultured in osteogenic medium (DMEM containing 10% FBS, 10 mmol/L β‐glycerophosphate, 0.1 μmol/L dexamethasone and 50 μmol/L ascorbic acid) and adipogenic medium (DMEM containing 10% FBS, 0.5 mmol/L isobutylmethylxanthine, 200 μmol/L indomethacin, 10 μmol/L bovine insulin and 1 μmol/L dexamethasone).

CCSMCs were isolated from the rat penises using a standard protocol in our laboratory.^18^ Then, the CCSMCs were cultured in DMEM supplemented with 10% FBS. The differential adhesion method was administered to minimize the number of other cells and to ensure that only CCSMCs were present in the culture flask. After 4 passages, the CCSMCs were identified by immunofluorescence detection of α‐smooth muscle actin (α‐SMA; 1:200; AF1032, Affinity Biosciences, USA) and desmin (1:200; AF5334, Affinity Biosciences).

### 2.3 Exosome isolation and characterization

Exosomes derived from cells were isolated from conventional culture medium. Briefly, after cells were cultured in exosome‐free medium for 48 hours, the cell supernatants were collected, and exosomes were isolated through multistep centrifugation. Dead cells and debris were eliminated through centrifugation at 300 × g for 10 minutes, 2000 × g for 20 minutes and 10 000 × g for 20 minutes. Then, each supernatant was centrifuged for 30 minutes at 1000 × g in a 100‐kDa MWCO ultrafiltration centrifuge tube (Amicon‐Ultra, Millipore, USA). Subsequently, each condensed supernatant was filtered through a 0.22‐μm filter, and a one‐fifth volume of ExoQuick reagent (EXOTC10A‐1, System Biosciences, USA) was added. Exosomes were isolated using ExoQuick reagent following the manufacturer's recommended protocol.^19^, ^20^

To characterize the exosomes, protein levels of CD9 (1:1000; AF5139, Affinity Biosciences), CD63 (1:1000; AF5117, Affinity Biosciences), TSG101 (1:1000; DF8427, Affinity Biosciences) and calnexin (1:1000; AF5362, Affinity Biosciences) were analysed through Western blot analysis. The presence and morphology of exosomes were observed using transmission electron microscopy (TEM, FEI Company, Netherlands).^21^ The size distribution and concentration of the exosomes were determined by nanoparticle tracking analysis using a nanoparticle tracking video microscope (ZetaView, Particle Metrix, Germany).^22^

### 2.4 Exosome labelling with PKH67 and uptake study

Based on their source, the exosomes were divided into the CCSMC group (CCSMC‐EXO), BMSC group (BMSC‐EXO) and ADSC group (ADSC‐EXO). To assess the in vitro uptake of CCSMC‐EXOs, BMSC‐EXOs and ADSC‐EXOs by smooth muscle cells, the purified exosomes were labelled with a PKH67 green fluorescent labelling kit (MINI67‐1KT, Sigma‐Aldrich, USA) according to the manufacturer's recommended procedure. Then, the labelled CCSMC‐EXOs, BMSC‐EXOs and ADSC‐EXOs were incubated with smooth muscle cells under normal conditions (cultured in DMEM only) and high‐glucose (HG) conditions (cultured in DMEM with 30 mmol/L glucose). Observations were conducted at 0, 4, 8, 16, 24 and 48 hours after incubation. At the end of each incubation period, the culture medium was discarded, and the cells were fixed with 4% paraformaldehyde. The slides were sealed with DAPI and photographed with a laser confocal microscope (SP8, Leica, Germany).

### 2.5 Animal experiments

To construct a diabetic rat model, streptozotocin (STZ; 60 mg/kg; Sigma‐Aldrich) was dissolved in vehicle (0.1 mol/L citrate phosphate buffer; pH = 4.2) and injected intraperitoneally into 50 rats. The same vehicle was injected intraperitoneally into the other 10 control rats (Con group, n = 10). The dose of the drug was based on that used in previous investigations by our laboratory.^5^, ^23^ Three and seven days after the intraperitoneal injection, tail‐vein blood glucose levels were gauged, and the onset of DM was defined as fasting glucose levels higher than 16.7 mmol/L at both measurements.

Twelve weeks later, 49 of the 50 rats that had been injected with streptozotocin had survived. Subsequently, an apomorphine (APO) test was administered to identify the rats with DMED, and rats with negative results were considered DMED rats according to the protocol in a previous study.^24^ Through the APO test, 31 rats were diagnosed with DMED and were further divided into four groups (DMED group, n = 7; DMED + CCSMC‐EXO group, n = 8; DMED + BMSC‐EXO group, n = 8; DMED + ADSC‐EXO group, n = 8). Three of the groups (the DMED + CCSMC‐EXO group, DMED + BMSC‐EXO group and DMED + ADSC‐EXO group) were injected with exosomes derived from different cell types. Exosomes were locally injected into the penis as described previously.^9^, ^25^ Briefly, 100 μg of exosomes labelled with DiR (D12731, Thermo Fisher Scientific, USA) and suspended in 200 μL of PBS were injected into the corpus cavernosum through intracavernous injection. Exosome accumulation and dynamics were tracked by in vivo fluorescence imaging at 0, 1, 2, 4, 24 hours and 6 days after injection using a Lago/Lago X Imager (Lago/Lago, Spectral Instruments Imaging, USA). The imaging data were calculated using AMIView 1.7.06 software (AMIView, Spectral Instruments Imaging, USA).

### 2.6 Evaluation of erectile function in vivo

Four weeks after intracavernous injection, the intracavernous pressure (ICP) was measured as previously described.^5^, ^23^ Briefly, under proper anaesthesia (100 mg/kg ketamine and 5 mg/kg midazolam), the major pelvic ganglion and cavernous nerves were exposed via midline laparotomy. A 25‐gauge needle was inserted into the left penile crus to monitor ICP. A PE‐50 tube full of heparinized saline (200 IU/mL) was cannulated into the left carotid artery, and the rat was connected to a signal acquisition system (AD Instruments PowerLab/4SP, Bella Vista, NSW, Australia) to continuously monitor mean arterial pressure (MAP). The cavernous nerves were electrically stimulated with a rustproof bipolar electrode (15.0 Hz; 5.0 V; duration 1 minutes) to elicit an erectile response. The ratio between the maximum increase in ICP and MAP (max ICP/MAP) was calculated to normalize variations in systemic blood pressure. Total ICP was represented by the area under the curve (AUC) during electrical stimulation.

When the evaluation of erectile function was over, the rats were killed by intraperitoneal injection of excess anaesthetic. Some corpus cavernosum tissue that had been pricked by the needle was discarded, and the rest was cut into four parts. One part was embedded in paraffin, and the other parts were stored in a −80°C freezer until subsequent experiments.

### 2.7 Western blotting

Tissue protein samples were prepared in RIPA buffer (Beyotime Biotechnology, Haimen, China) containing phosphatase inhibitor and protease inhibitor cocktails (Roche Applied Science, Indianapolis, IN, USA). Samples containing 20 μg of protein were subjected to sodium dodecyl sulphate‐polyacrylamide gel electrophoresis, and the proteins were then transferred to a polyvinylidene difluoride membrane. The membrane was blocked with Tris‐buffered saline‐Tween with 5% bovine serum albumin and incubated at 4°C overnight with primary antibodies against α‐SMA (1:1000; AF1032, Affinity Biosciences), Collagen I (1:1000; AF7001, Affinity Biosciences), TGF‐β1 (1:1000; AF1027, Affinity Biosciences), eNOS (1:1000; AF0096, Affinity Biosciences), nNOS (1:1000; AF6249, Affinity Biosciences) or β‐actin (1:1000; 20536‐1‐AP, Proteintech, China). After hybridization of secondary antibodies (1:5000 or 1:10000; ProMab, USA), the immunoreactive proteins were detected through an enhanced chemiluminescence detection system (Pierce, Thermo Fisher Scientific, Rockford, IL, USA).

### 2.8 Immunofluorescence staining

Penile tissue sections were dewaxed in dimethylbenzene and washed with PBS. The slides were blocked and incubated with primary antibodies against eNOS (1:200; Affinity Biosciences) and nNOS (1:200; Affinity Biosciences). Subsequently, slides were incubated with the appropriate secondary antibodies. DAPI (4',6‐diamino‐2‐phenylindole; Sigma‐Aldrich, St. Louis, MO, USA) was used to stain the cell nuclei. Digital images were obtained using an Olympus BX51 fluorescence microscope (Olympus Corporation, Tokyo, Japan).

### 2.9 Immunohistochemistry and Masson's trichrome staining

Penile tissue sections were processed for immunohistochemical investigations and incubated with antibody against TGF‐β1 (1:100; Affinity Biosciences), eNOS (1:100; Affinity Biosciences) or nNOS (1:100; Affinity Biosciences) at 37°C for 1 hour. The sections were then incubated with biotinylated secondary antibodies. Masson's trichrome staining was carried out to determine the ratio between smooth muscle and collagen in the corpus cavernosum as previously described.^26^

### 2.10 Analysis of nitric oxide (NO) and cyclic guanosine monophosphate (cGMP) levels

The NO levels were determined using a nitrate‐nitrite assay kit (S0024, Beyotime Biotechnology, China), and the cGMP levels were detected using an enzyme‐linked immunosorbent assay kit (F15182, R & D Systems, Minneapolis, MN, USA). All steps were carried out according to the manufacturer's recommended protocol. The NO and cGMP levels were normalized to the protein concentration.

### 2.11 Statistical analyses

The images were analysed using Image‐Pro Plus 6.0 software (Media Cybernetics, Silver Spring, MD, USA), and data were analysed using GraphPad Prism 7.0 (GraphPad Software, San Diego, CA, USA). The results are expressed as the mean plus or minus the standard deviation. Multiple groups were compared using one‐way analysis of variance followed by the Tukey‐Kramer test for post hoc comparisons. Statistical significance was set at *P* < .05.

## 3 RESULTS

### 3.1 Cell identification and exosome characterization

**FIGURE 1:**
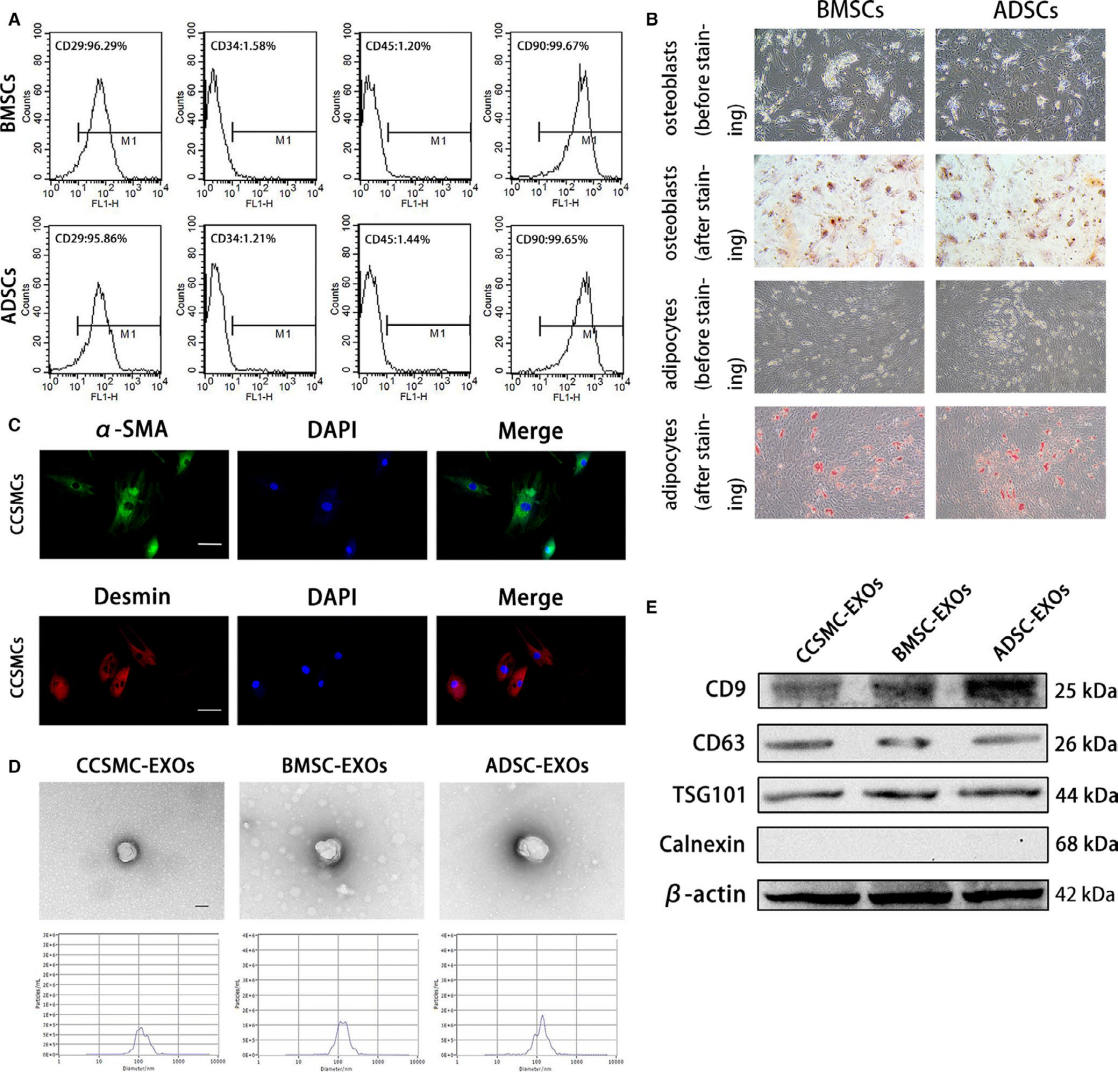
Cell identification and exosome characterization. (A) Representative flow cytometry histograms of BMSCs and ADSCs show positive staining for CD29 and CD90 but not for CD34 and CD45. (B) BMSCs and ADSCs were successfully induced into osteoblasts (positively stained with Alizarin Red S) and adipocytes (positively stained with Oil Red O). The magnification is 100×. (C) Representative immunofluorescence results of CCSMCs show positive expression for α‐SMA and desmin. Scale bars = 50 μm. (D) Exosomes derived from CCSMCs, BMSCs and ADSCs were observed using transmission electron microscopy, and the particle size distributions of the exosomes were measured by nanoparticle tracking analysis. Scale bars = 100 nm. (E) Representative results of Western blot analysis of exosomes derived from CCSMCs, BMSCs and ADSCs show positive expression for CD9, CD63 and TSG101 but not for calnexin. CCSMC: corpus cavernosum smooth muscle cell; BMSC: bone marrow stem cell; ADSC: adipose‐derived stem cell; CCSMC‐EXOs: exosomes derived from corpus cavernosum smooth muscle cells; BMSC‐EXOs: exosomes derived from bone marrow stem cells; ADSC‐EXOs: exosomes derived from adipose‐derived stem cells; α‐SMA: α‐smooth muscle actin; DAPI: 4’,6‐diamidino‐2‐phenylindole

To identify the MSCs (BMSCs and ADSCs) used in this study, we first analysed the expression of cell surface antigens. As shown in Figure 1A, the cells expressed the MSC markers CD29 and CD90 but not the endothelial or hematopoietic markers CD34 and CD45. Meanwhile, Figure 1B shows that the MSCs were successfully induced into osteoblasts (stained positive with Alizarin Red S) and adipocytes (stained positive with Oil Red O), which indicates the differentiation potential of the MSCs. To identify the CCSMCs used in this study, we subjected cells isolated from the rat penises to immunofluorescence staining for the specific markers of smooth muscle cells α‐SMA and desmin. The results in Figure 1C show positive staining for α‐SMA and desmin, and the proportion of cells with positive staining is greater than 95%, indicating that the cultured cells were CCSMCs.

The isolated exosomes were visualized and detected using TEM. As shown, the exosomes were approximately 100 nm in diameter (Figure 1D). Meanwhile, nanoparticle tracking analysis indicated that the diameters of typical particles were mainly approximately 100 nm (Figure 1D). Furthermore, the Western blot analysis results in Figure 1E revealed that the exosomal markers CD9, CD63 and TSG101 were abundant in our isolated exosomes, while staining for calnexin was negative. All these results indicate that the isolation of exosomes was successful.

### 3.2 Exosomes derived from CCSMCs were more easily taken up by CCSMCs

**FIGURE 2:**
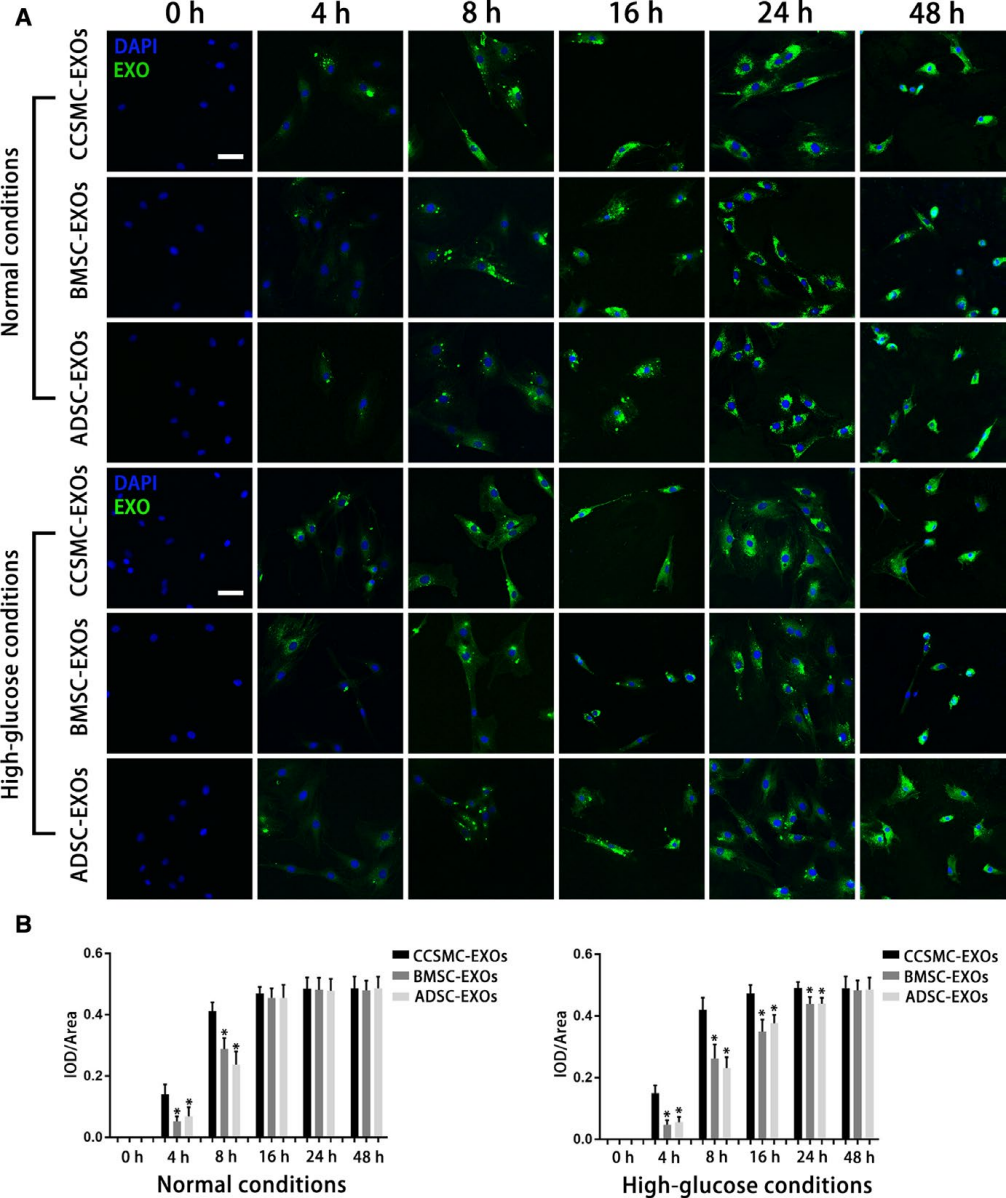
Uptake of exosomes by CCSMCs. (A) Representative confocal images of PKH67‐labelled exosomes taken up by CCSMCs cultured under different conditions are shown. The culture time is indicated. Scale bars = 50 μm. (B) Time courses of the uptake of different types of exosomes by CCSMCs under different conditions are presented as bar graphs. Data are expressed as the mean ± standard deviation. ^*^ *P* < .05 compared with the CCSMC‐EXO group. CCSMC‐EXOs: exosomes derived from corpus cavernosum smooth muscle cells; BMSCs‐EXOs: exosomes derived from bone marrow stem cells; ADSC‐EXOs: exosomes derived from adipose‐derived stem cells; DAPI: 4',6‐diamidino‐2‐phenylindole; IOD: integral optical density; IOD/area: mean optical density

To determine the time course of the uptake of different types of exosomes by CCSMCs under different conditions, we labelled CCSMC‐EXOs, BMSC‐EXOs and ADSC‐EXOs with a green fluorescent marker, PKH67, and incubated them with CCSMCs under normal conditions and high‐glucose conditions. As shown in Figure 2A and B, in both normal and high‐glucose environments, a significantly greater number of CCSMC‐EXOs than BMSC‐EXOs and ADSC‐EXOs were taken up by the cells after 4 and 8 hours of culture with CCSMCs (*P* < .05). Under normal conditions, the uptake of the three types of exosomes by the cells reached the same level after approximately 16 hours of culture, and there was no significant difference in uptake of the exosomes at 24 and 48 hours (*P* > .05). Under high‐glucose conditions, the uptake of CCSMC‐EXOs at 16 and 24 hours remained higher than that of BMSC‐EXOs and ADSC‐EXOs (*P* < .05). Subsequently, the uptake of the three types of exosomes by the cells reached the same level after approximately 48 hours of culture (*P* > .05). The above results suggest that CCSMC‐EXOs were more easily taken up by CCSMCs.

### 3.3 Exosomes derived from CCSMCs were more easily retained in the corpus cavernosum of DMED rats

**FIGURE 3:**
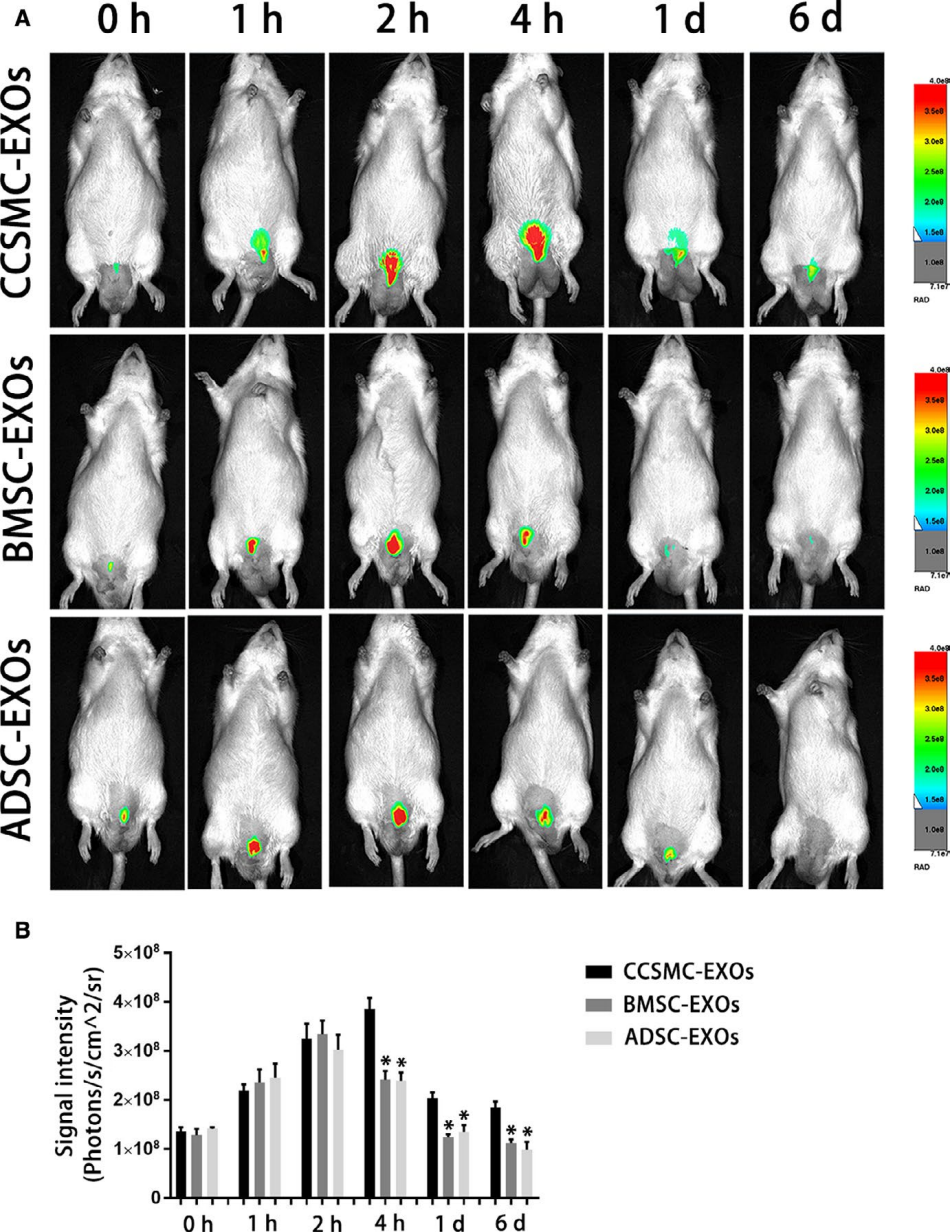
Exosome intracavernous injection and in vivo imaging. (A) Representative images of DMED rats that underwent intracavernous injection with DiR‐labelled exosomes. (B) The signal intensities of different DiR‐labelled exosomes in DMED rats at different time points after injection are presented as a bar graph. Data are expressed as the mean ± standard deviation. ^*^ *P* < .05 compared with the CCSMC‐EXO group. CCSMC‐EXOs: exosomes derived from corpus cavernosum smooth muscle cells; BMSC‐EXOs: exosomes derived from bone marrow stem cells; ADSC‐EXOs: exosomes derived from adipose‐derived stem cells; DMED: diabetes mellitus‐induced erectile dysfunction

To compare the retention time of different types of exosomes in DMED rats after their intracavernous injection, CCSMC‐EXOs, BMSC‐EXOs and ADSC‐EXOs were labelled with DiR, and the signal intensities of the exosomes (used to represent the amount of exosomes) at 0, 1, 2, 4 hours, 1 and 6 days after injection were observed by in vivo fluorescence imaging. As shown in Figure 3A and B, the peak signal intensity for CCSMC‐EXOs approximately 4 hours after injection was higher than that for the other two types of exosomes (*P* < .05). Subsequently, CCSMC‐EXOs were maintained at a certain level in the corpus cavernosum at 1 day and 1 week after injection, and the level of CCSMC‐EXOs was significantly higher than that of the other two types of exosomes (*P* < .05). The above results suggest that compared with BMSC‐EXOs and ADSC‐EXOs, CCSMC‐EXOs were more easily retained in the corpus cavernosum of DMED rats.

### 3.4 Exosomes derived from CCSMCs could improve erectile function in DMED rats

**FIGURE 4:**
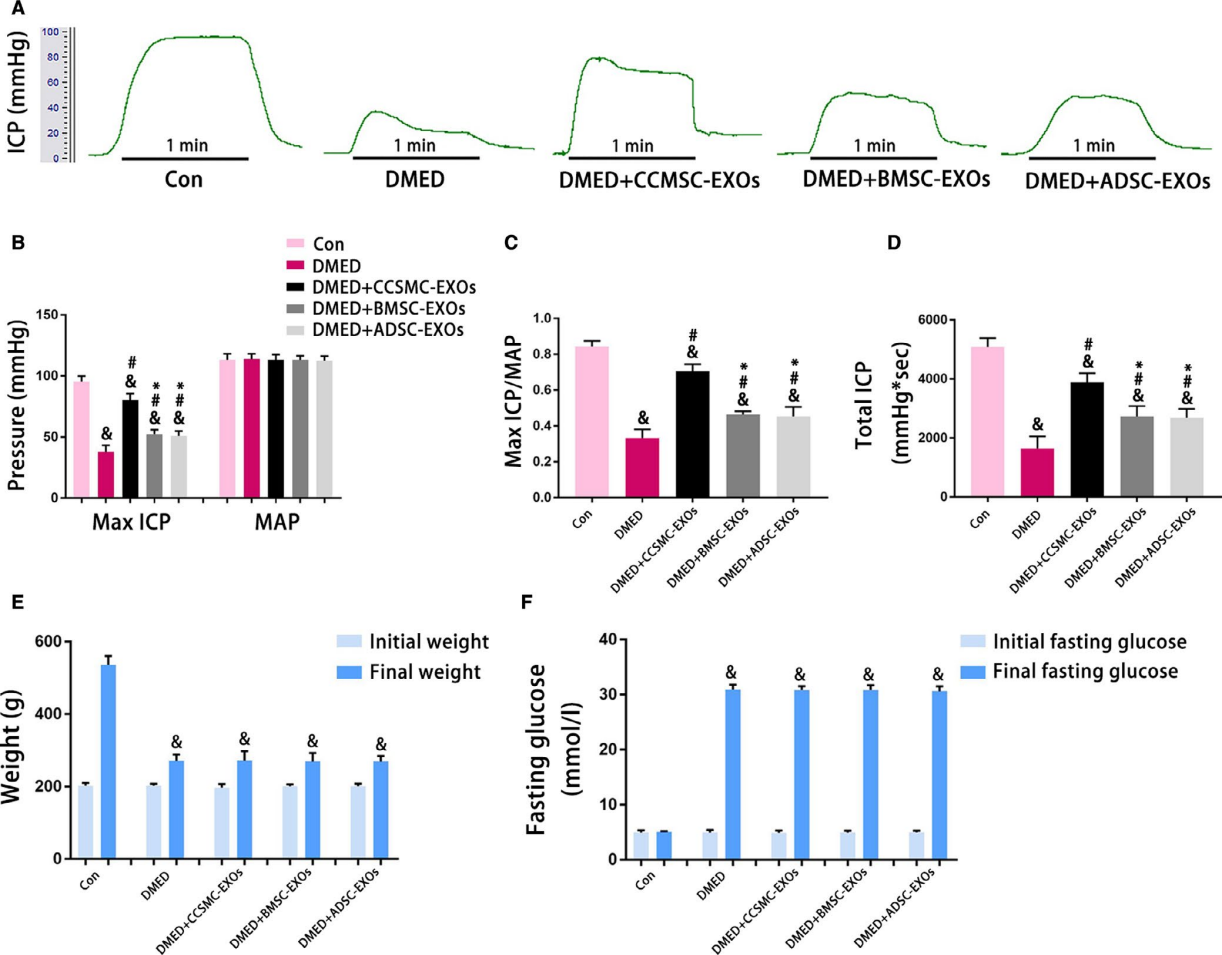
Evaluation of erectile function and metabolic indexes. (A) Representative ICP traces measured by stimulating the cavernous nerves with 5 V for 1 min. (B) The max ICP and MAP values for each group are presented as a bar graph. (C) The max ICP/MAP values for each group are presented as a bar graph to show the erectile function. (D) The total ICP values for each group are presented as a bar graph to show the erectile function. (E) Initial and final weights for each group are presented as a bar graph. (F) Initial and final fasting glucose levels for each group are presented as a bar graph. Data are expressed as the mean ± standard deviation. ^&^ *P* < .05 compared with the Con group. ^#^ *P* < .05 compared with the DMED group. ^*^ *P* < .05 compared with the DMED + CCSMC‐EXO group. CCSMC‐EXOs: exosomes derived from corpus cavernosum smooth muscle cells; BMSC‐EXOs: exosomes derived from bone marrow stem cells; ADSC‐EXOs: exosomes derived from adipose‐derived stem cells; DMED: diabetes mellitus‐induced erectile dysfunction; ICP: intracavernous pressure; MAP: mean arterial pressure

First, as shown in Figure 4E and F, no significant differences in the initial weights and initial fasting glucose levels between rats in each group were observed (*P* > .05). Nevertheless, at 16 weeks after diabetes induction, the final weight of the Con group was obviously higher than that of other groups (*P* < .05). In contrast, the final fasting glucose level of the Con group was significantly lower than that of other groups (*P* < .05).

Second, as shown in Figure 4A to D, max ICP/MAP and total ICP were used to evaluate erectile function in the rats. These indicators were significantly reduced in the DMED group and partly increased after administration of the different types of exosomes, although they were still lower than those in the Con group (*P* < .05). Compared with the injection of BMSC‐EXOs and ADSC‐EXOs, the injection of CCSMC‐EXOs more effectively improved erectile function (*P* < .05). The above results indicate that CCSMC‐EXOs could effectively improve erectile function in DMED rats.

### 3.5 Exosomes derived from CCSMCs could inhibit corporal fibrosis in the corpus cavernosum of DMED rats

**FIGURE 5:**
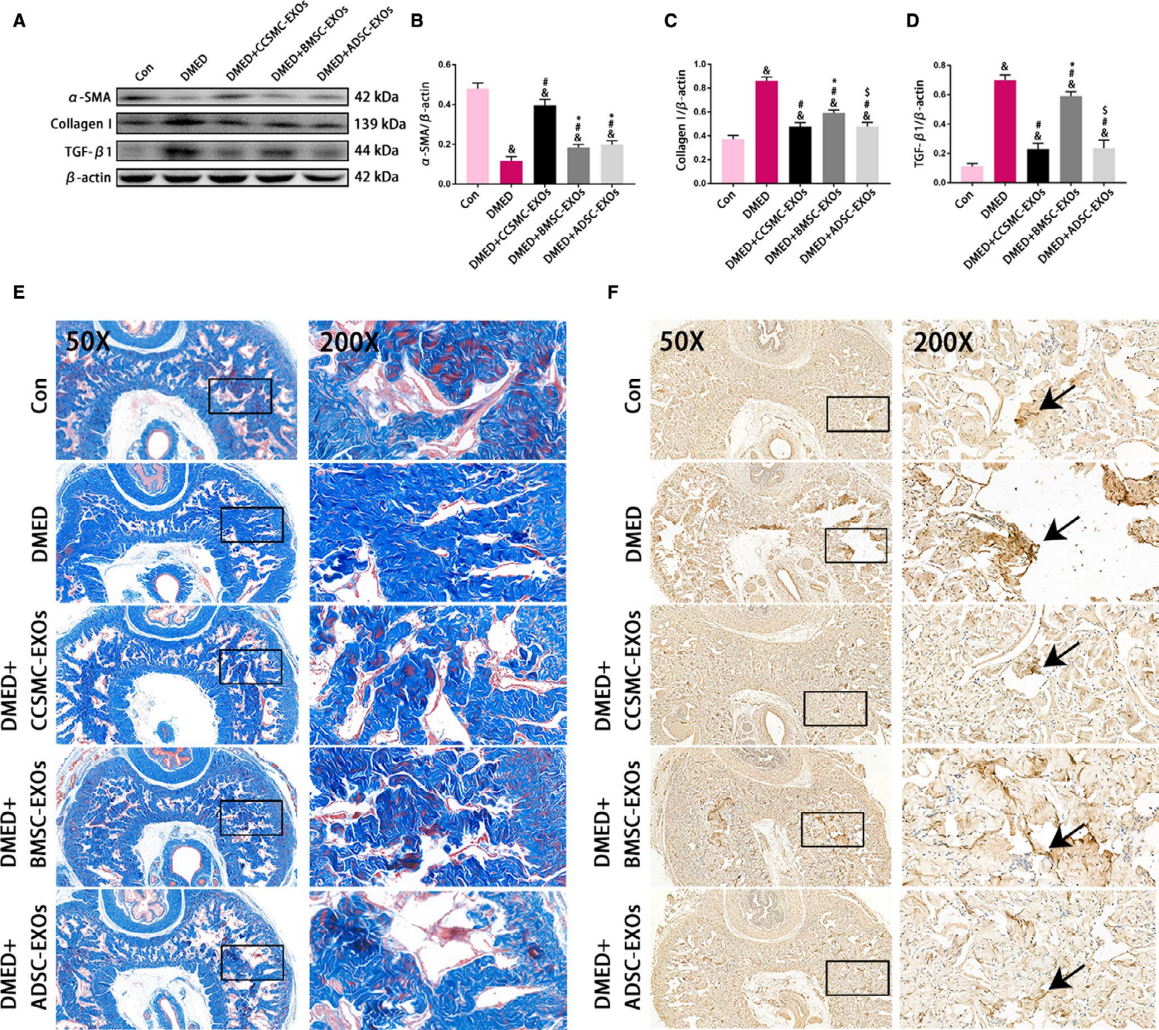
Evaluation of corporal fibrosis in the corpus cavernosum. (A) Representative results of Western blot analysis of α‐SMA, Collagen I and TGF‐β1 in rats from all five groups. (B) Expression of α‐SMA in all five groups with β‐actin as the loading control is presented as a bar graph. (C) Expression of Collagen I in all five groups with β‐actin as the loading control is presented as a bar graph. (D) Expression of TGF‐β1 in all five groups with β‐actin as the loading control is presented as a bar graph. (E) Representative images showing smooth muscle and collagen staining in all five groups. Smooth muscle and collagen in the corpus cavernosum are stained red and blue, respectively. Original magnification, 50× and 200×. (F) Representative results of immunohistochemistry for TGF‐β1 in all five groups (50× and 200×). The arrows indicate TGF‐β1 expression. Data are expressed as the mean ± standard deviation. ^&^ *P* < .05 compared with the Con group. ^#^ *P* < .05 compared with the DMED group. ^*^ *P* < .05 compared with the DMED + CCSMC‐EXO group. ^$^ *P* < .05 compared with the DMED + BMSC‐EXO group. CCSMC‐EXOs: exosomes derived from corpus cavernosum smooth muscle cells; BMSC‐EXOs: exosomes derived from bone marrow stem cells; ADSC‐EXOs: exosomes derived from adipose‐derived stem cells; DMED: diabetes mellitus‐induced erectile dysfunction

As shown in Figure 5A‐C,E, diabetes caused corporal fibrosis in the corpus cavernosum, as demonstrated by a decrease in the smooth muscle content and increase in collagen content in the DMED group compared to the Con group (*P* < .05). All exosome treatment groups exhibited partial inhibition of corporal fibrosis according to the results of Western blot analysis and Masson's trichrome staining. Importantly, compared with BMSC‐EXOs and ADSC‐EXOs, CCSMC‐EXOs increased the smooth muscle content to a greater extent (*P* < .05). TGF‐β1, an important profibrotic factor in the corpus cavernosum, was significantly increased in the DMED group. However, the level of TGF‐β1 in the CCSMC‐EXO group and ADSC‐EXO group was significantly lower than that in the DMED group, although it was still higher than that in the Con group (*P* < .05; Figure 5A,D,F). The above results indicate that CCSMC‐EXOs could inhibit corporal fibrosis in the corpus cavernosum of DMED rats.

### 3.6 Exosomes derived from CCSMCs could improve the NO/cGMP signalling pathway in the corpus cavernosum of DMED rats

**FIGURE 6:**
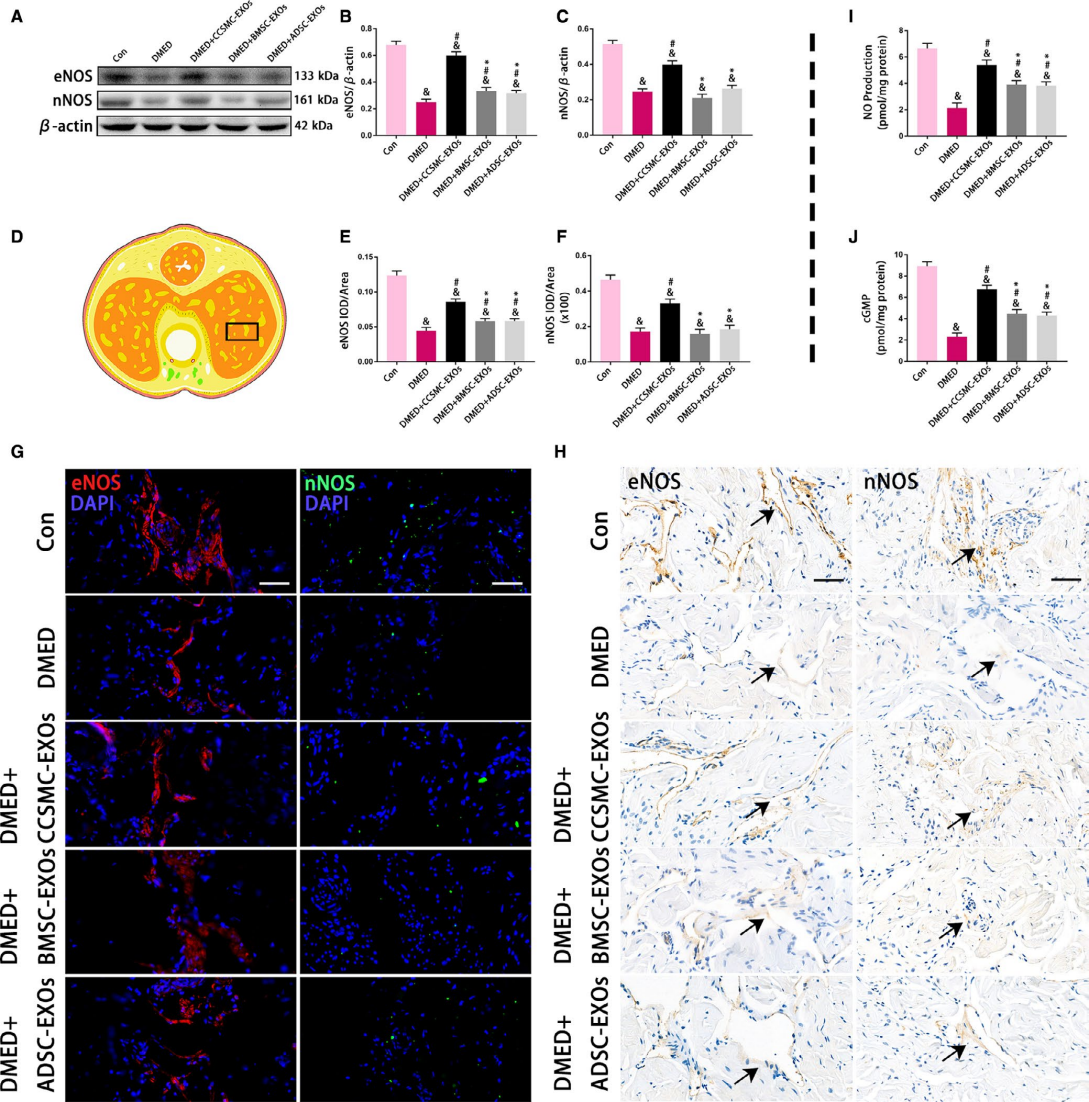
eNOS and nNOS expression with the NO and cGMP concentration in the corpus cavernosum. (A) Representative results of Western blot analysis of eNOS and nNOS in the rats of all five groups. (B) Expression of eNOS in all five groups with β‐actin as the loading control is presented as a bar graph. (C) Expression of nNOS in all five groups with β‐actin as the loading control is presented as a bar graph. (D) The black rectangle indicates the area of the corpus cavernosum selected for comparison. (E) The eNOS level is defined as eNOS IOD/area according to the immunofluorescence results. (F) The nNOS level is defined as nNOS IOD/area according to the immunofluorescence results. (G) Representative results of immunofluorescence analysis of eNOS and nNOS in all five groups. Scale bars = 50 μm. (H) Representative results of immunohistochemistry analysis of eNOS and nNOS in all five groups. The arrows indicate eNOS and nNOS expression (Because the expression levels of eNOS and nNOS in DMED group were very low, the coloring was very light). Scale bars = 50 μm. (I) The NO production in all five groups was determined with an assay kit. (J) The cGMP concentration in all five groups was determined with an assay kit. Data are expressed as the mean ± standard deviation. ^&^ *P* < .05 compared with the Con group. ^#^ *P* < .05 compared with the DMED group. ^*^ *P* < .05 compared with the DMED + CCSMC‐EXO group. CCSMC‐EXOs: exosomes derived from corpus cavernosum smooth muscle cells; BMSC‐EXOs: exosomes derived from bone marrow stem cells; ADSC‐EXOs: exosomes derived from adipose‐derived stem cells; DMED: diabetes mellitus‐induced erectile dysfunction; DAPI: 4',6‐diamidino‐2‐phenylindole; eNOS: endothelial nitric oxide synthase; nNOS: neuronal nitric oxide synthase; NO: nitric oxide; cGMP: cyclic guanosine monophosphate; IOD: integral optical density; IOD/area: mean optical density

Diabetes exerted a significant inhibitory effect on the NO/cGMP signalling pathway, and exosome treatment improved this pathway. To better understand this process, endothelial nitric oxide synthase (eNOS) and neuronal nitric oxide synthase (nNOS), the two most important factors that produce NO in the corpus cavernosum, were detected using Western blot analysis and immunol staining. As shown in Figure 6A‐C, the results of Western blot analysis revealed that the levels of eNOS and nNOS were higher in the Con group than in the other four groups. Although the levels of eNOS and nNOS in the DMED + CCSMC‐EXO group were lower than those in the Con group, they were significantly higher than those in the other DMED groups (*P* < .05). Consistent with the Western blot results, the results of immunol staining for eNOS and nNOS also showed decreased eNOS and nNOS expression in the DMED group, which was partly increased after CCSMC‐EXO treatment (*P* < .05; Figure 6D to H).

In addition, NO and cGMP were directly detected using an assay kit. The NO and cGMP concentrations were obviously reduced in the DMED group compared with the other four groups and partly increased after treatment with exosomes. Furthermore, compared with BMSC‐EXOs and ADSC‐EXOs, CCSMC‐EXOs increased the concentrations of NO and cGMP to a greater extent (*P* < .05; Figure 6I and J). All the above indicators show that CCSMC‐EXOs could improve the NO/cGMP signalling pathway in the corpus cavernosum of DMED rats.

## 4 DISCUSSION

As one of the most common types of ED caused by medical problems, DMED still lack effective and specific treatments.^27^ Recent studies have confirmed that paracrine therapy is an important method by which MSCs treat ED.^28^ As a crucial component of paracrine secretion, exosomes are a kind of bioactive substance that participates in many physiologic and pathologic processes, such as antifibrosis processes, intercellular communication and tissue regeneration.^29^, ^30^, ^31^ MSC‐EXOs were reported to partially ameliorate DMED.^8^, ^9^ However, many unknown issues worth exploring, such as the retention effect of MSC‐EXOs in the corpus cavernosum and whether other effective and specific exosomes can be used for the treatment of DMED, remained. In our current study, by comparing CCSMC‐EXOs with MSC‐EXOs, we initially demonstrated that CCSMC‐EXOs could effectively improve erectile function in diabetic rats. More specifically, our experimental results showed that CCSMC‐EXOs could be targeted for enrichment in CCSMCs. After intracavernous injection, CCSMC‐EXOs remained in the corpus cavernosum and improved erectile function by inhibiting corporal fibrosis and improving the NO/cGMP signalling pathway, indicating that CCSMC‐EXOs could be a novel exosome‐based therapeutic strategy for DMED.

Exosomes can be taken up by cells through unspecific pinocytosis or specific molecular interactions, including membrane‐exposed sugars, lipids or proteins.^32^ A breakthrough in understanding exosome transmission between cells, exosomes were discovered to express a certain number of parent cell‐derived markers.^33^, ^34^, ^35^ This means that cells might be more inclined to take up exosomes of the same cell origin. Alvarez‐Erviti L et al reported similar conclusions; they found that exosomes have the unique property of homing selectivity because they can express cell type‐specific protein markers discovered in the membrane of the parent cell.^36^ In our research, we incubated labelled CCSMC‐EXOs and MSC‐EXOs with CCSMCs and then observed uptake of the exosomes at different time points using laser confocal microscopy. We found that CCSMC‐EXOs could be taken up by CCSMCs faster than MSC‐EXOs in normal and high‐glucose environments. This suggests that CCSMC‐EXOs might have a faster onset time as a potential therapeutic drug for DMED. However, this finding needs to be further confirmed by more studies. Subsequently, consistent with the results of the in vitro uptake study, our in vivo imaging results showed that intracavernous injection of DiR‐labelled CCSMC‐EXOs was associated with a relatively high peak concentration and long retention time. The exact mechanism by which CCSMC‐EXOs were more easily retained in the corpus cavernosum of DMED rats is unclear and needs to be further elucidated in future studies. One possible hypothesis is that CCSMCs can take up CCSMC‐EXOs faster, which effectively reduces the loss of CCSMC‐EXOs injected into the corpus cavernosum. As reported, because of the anatomical characteristics of the corpus cavernosum, locally injected fluid is not easily retained in the corpus cavernosum for a long time.^37^ In summary, our data are the first demonstration of the dynamics of CCSMC‐EXOs injected in the corpus cavernosum and thus provide important information regarding the administration of CCSMC‐EXOs in exosome‐based therapy for DMED.

Erectile dysfunction is typically observed in patients with vasculogenic, neurogenic or endocrinologic diseases and is one of the common early complications of diabetes.^3^, ^4^ After successfully establishing a rat model of diabetes, we found that the erectile function of diabetic rats was seriously impaired, which is consistent with observations in diabetic men with ED. Subsequently, we observed that CCSMC‐EXOs or MSC‐EXOs could preserve the erectile function of DMED rats. The therapeutic effect of MSC‐EXOs on DMED has been reported in the relevant literature,^8^, ^9^ and our results are consistent with the conclusions reported in these studies. Furthermore, for the first time, our study demonstrates the therapeutic effect of CCSMC‐EXOs on DMED.

We found that the specific mechanism of this therapeutic effect might be related to the ability of CCSMC‐EXOs to inhibit corporal fibrosis, as shown by the results of immunohistochemistry and Masson's trichrome staining. Our previous research and other studies have shown that corporal fibrosis caused by reduced corpus cavernosum smooth muscle content and increased collagen content is one of the main mechanisms of DMED.^5^, ^38^ Here, we observed a dramatic decrease in the smooth muscle content and a significant increase in the collagen content of the corpus cavernosum of DMED rats, and treatment with CCSMC‐EXOs or MSC‐EXOs effectively alleviated these histopathological changes in the corpus cavernosum. On the one hand, our data support previous reports that MSC‐EXOs have an inhibitory effect on corporal fibrosis,^28^ and on the other hand, they also indicate that CCSMC‐EXOs have a more obvious inhibitory effect on corporal fibrosis than other types of exosomes do. We believe that this inhibition is related to the ability of CCSMC‐EXOs to effectively reduce the expression of TGF‐β1. TGF‐β1 is an important profibrotic cytokine that has been recognized as the key factor involved in the formation and development of corporal fibrosis.^39^ Kim et al reported that the increased expression of TGF‐β1 is closely related to the accumulation and deposition of collagen.^40^ We found that compared with BMSC‐EXOs, CCSMC‐EXOs could significantly reduce the expression of TGF‐β1, thereby reducing the collagen content. This partly explains why CCSMC‐EXOs can inhibit corporal fibrosis. Interestingly, in our study, ADSC‐EXOs and CCSMC‐EXOs had similar effects in reducing TGF‐β1 expression. However, CCSMC‐EXOs could significantly increase the expression of α‐SMA, which means that CCSMC‐EXOs can not only effectively reduce the collagen content but also significantly increase the smooth muscle content. Hence, CCSMC‐EXOs have an obvious inhibitory effect on corporal fibrosis.

Another important effect of CCSMC‐EXOs may be their improvement of the NO/cGMP signalling pathway. The NO/cGMP signalling pathway is the most important pathway known to regulate penile erection, and a well‐recognized theory postulates that down‐regulation of this pathway contributes to DMED.^41^ In the NO/cGMP signalling pathway, NO produced by eNOS in cavernous endothelial cells and nNOS in cavernous nerves induce relaxation of the corpus cavernosum smooth muscle by increasing the cGMP content in CCSMCs, thereby inducing erection.^42^, ^43^ In our study, we found that CCSMC‐EXOs could up‐regulate the expression of eNOS and nNOS in the corpus cavernosum of diabetic rats, thereby increasing the levels of NO and cGMP. The role of exosomes as important regulators between vascular smooth muscle cells and vascular endothelial cells that maintain vascular homeostasis has been highlighted by recent studies.^44^, ^45^ Similarly, our research indicates that CCSMC‐EXOs might be an important component of intercellular communication between smooth muscle cells and endothelial cells in the corpus cavernosum and might play a role in repairing cavernous endothelial cells damaged by diabetes. Recently, accumulated evidence has shown that exosomes from an increasing number of cell types, such as Schwann cells and macrophages, can promote nerve regeneration.^46^ As a supplement to the above literature, our data provide evidence for the nerve‐regeneration effect of CCSMC‐EXOs in the corpus cavernosum of diabetic rats. Interestingly, it was reported that MSC‐EXOs could increase the expression of nNOS in the corpus cavernosum in a rat model of cavernous nerve injury,^47^, ^48^ but no similar phenomenon was observed in our diabetic rat model. We think this may be because of the difference in animal models, which should be confirmed by more studies in the future. In summary, our data indicate that CCSMC‐EXOs can ameliorate DMED by inhibiting fibrosis and stimulating NO/cGMP pathway. As we all know, exosomes contain various substances, of which microRNAs and proteins may be important factors that mediate the therapeutic effects of exosomes.^49^ Zhu et al reported that exosomes from ADSC can ameliorate DMED by delivering microRNAs, including some proangiogenic microRNAs (miR‐126, miR‐130a and miR‐132) and antifibrotic microRNAs (miR‐let7b and miR‐let7c).^8^ In addition, Wang et al reported that corin in exosomes from ADSC also has the effect of ameliorating DMED.^50^ At present, there is no relevant report on CCSMC‐EXOs. We speculate that CCSMC‐EXOs may also ameliorate DMED through specific microRNAs and proteins, which should be confirmed by more studies in the future.

There are several limitations to our current study. First, we studied the role of CCSMC‐EXOs in type 1 diabetes, whereas the effectiveness of CCSMC‐EXOs in type 2 diabetes is not yet clear. Currently, type 2 diabetes is more common than type 1 diabetes. Although the pathological changes of ED in the corpus cavernosum caused by type 1 diabetes and type 2 diabetes are similar, the role of CCSMC‐EXOs in type 2 diabetes remains to be determined. Second, among the several isolation methods for CCSMC‐EXOs, we used the ExoQuick method, which is a widely accepted method of exosome isolation. This method can achieve high yield and quality, but its specificity is relatively low.^51^ Therefore, additional studies using other isolation methods are necessary to verify our results. Third, we used a single dose (100 μg) of exosomes for intracavernous injection. Although this is a common dose used in exosome‐related in vivo studies, future studies will be conducted to identify the optimum dose. Fourth, it is still a challenge to fully understand the specific composition of exosomes.^52^ we found that ADSC‐EXOs, BMSC‐EXOs and CCSMC‐EXOs were different in dynamics and functions. The main reason for these differences is that the cargo of exosomes from different cells are different. However, we do not yet know the specific differences in the cargo of these exosomes, which needs to be further studied. Finally, we observed that CCSMC‐EXOs could effectively remain in the corpus cavernosum and improve erectile function by inhibiting corporal fibrosis and up‐regulating the NO/cGMP signalling pathway, but the exact mechanism has not been fully elucidated. Unknown microRNAs or proteins may participate in this complex process, which our laboratory has recently initiated research to further clarify.

In conclusion, our work initially identified the possible role of CCSMC‐EXOs in ameliorating DMED through inhibiting corporal fibrosis and modulating the NO/cGMP signalling pathway. We also uncovered the dynamics of injected exosomes in the corpus cavernosum and compared the therapeutic effects of CCSMC‐EXOs and MSC‐EXOs on DMED. These findings may provide new insights into the role of exosomes in the treatment of DMED.

## CONFLICT OF INTEREST

The authors confirm that there are no conflict of interest.

## AUTHOR CONTRIBUTIONS

Jingyu Song and Jihong Liu: Design. Jingyu Song and Taotao Sun: Data acquisition, data interpretation and analysis, and drafting and revising. Zhe Tang, Yajun Ruan and Kang Liu: Study material contribution. Ke Rao, Ruzhu Lan and Shaogang Wang: Reading and correcting. Tao Wang and Jihong Liu: Experimental direction and drafting. All authors: Reading and approval.

## Data Availability

The data that support the findings of this study are available from the corresponding author upon reasonable request.
